# Zinc Oxide Nanoparticles Application Alleviates Arsenic (As) Toxicity in Soybean Plants by Restricting the Uptake of as and Modulating Key Biochemical Attributes, Antioxidant Enzymes, Ascorbate-Glutathione Cycle and Glyoxalase System

**DOI:** 10.3390/plants9070825

**Published:** 2020-06-30

**Authors:** Parvaiz Ahmad, Mohammed Nasser Alyemeni, Asma A. Al-Huqail, Moneerah A. Alqahtani, Leonard Wijaya, Muhammad Ashraf, Cengiz Kaya, Andrzej Bajguz

**Affiliations:** 1 Botany and Microbiology Department, College of Science, King Saud University, 11451 Riyadh, Saudi Arabia; mnyemeni@ksu.edu.sa (M.N.A.); aalhuqail@ksu.edu.sa (A.A.A.-H.); 439203051@student.ksu.edu.sa (M.A.A.); leon077@gmail.com (L.W.); 2 University of Agriculture Faisalabad, Faisalabad 38040, Pakistan; ashrafbot@yahoo.com; 3 Soil Science and Plant Nutrition Department, Agriculture Faculty, Harran University, 63210 Sanliurfa, Turkey; c_kaya70@yahoo.com; 4 Department of Biology and Ecology of Plants, Faculty of Biology, University of Bialystok, 15-245 Bialystok, Poland; abajguz@uwb.edu.pl

**Keywords:** arsenic stress, soybean, growth, antioxidant enzymes, ascorbate–glutathione cycle, glyoxalase system

## Abstract

Accumulation of arsenic (As) in soils is increasing consistently day-by-day, which has resulted in increased toxicity of this element in various crop plants. Arsenic interferes with several plant metabolic processes at molecular, biochemical and physiological levels, which result in reduced plant productivity. Hence, the introduction of novel ameliorating agents to combat this situation is the need of the hour. The present study was designed to examine the effect of zinc oxide nanoparticles (ZnO–NPs) in As-stressed soybean plants. Various plant growth factors and enzymes were studied at varying concentrations of As and ZnO–NPs. Our results showed that with the application of ZnO–NPs, As concentration declined in both root and shoot of soybean plants. The lengths of shoot and root, net photosynthetic rate, transpiration, stomatal conductance, photochemical yield and other factors declined with an increase in external As level. However, the application of ZnO–NPs to the As-stressed soybean plants resulted in a considerable increase in these factors. Moreover, the enzymes involved in the ascorbate–glutathione cycle including superoxide dismutase (SOD), catalase (CAT), ascorbate peroxidase (APX) and glutathione reductase (GR) showed a significant increase in their activity with the application of ZnO–NPs to the As-stressed plants. Hence, our study confirms the significance of ZnO–NPs in alleviating the toxicity of As in soybean plants.

## 1. Introduction

Arsenic (As) is present in lithosphere and is considered as one of the major environmental contaminants [1]. The main As pollution is due to the anthropogenic activities like, over-use of herbicides and pesticides, combustion of coal and preservation of timber (Sharma, 2013). Soil parameters such as redox state and pH have a foremost impact on As toxicity due to its altered accessibility (solubility and mobility) to plants [2]. Uptake of As is believed to be affected by some factors such as pH, nutrient supply, soil type and mugineic acid excreted by some grassy plants [2]. Arsenic within plant cells alters normal cellular metabolic activities mostly by way of binding to the enzymes and changing their course of action, interfering with carbon and sulfur metabolism and disruption of nitrogen assimilation [3]. Arsenic (As III) can react with sulfhydryl groups of proteins and enzymes which can cause loss of function and even cell death [4]. Under higher concentrations of arsenic, the plant fails to balance between the resistance and toxicity, resulting in plant death. [5]. Arsenic stress can significantly reduce the rate of photosynthesis, transpiration as well as stomatal conductance in plants [4] which could be ascribed to metal-induced reduction of water transport [6]. Arsenic toxicity also leads to oxidative stress by the overproduction of reactive oxygen species (ROS) in plants [7,8,9]. Arsenic stress also enhances the production of methyl glyoxalate (MG) which can damage the normal functioning of the plant cell [10]. According to Yadav, et al. [11], MG and ROS attack the biomolecules and alter their basic structure as well as functions. To mitigate this heavy metal-induced toxicities, plants have developed different stress tolerance mechanisms. The synthesis of osmolytes (proline and glycine betaine (GB) was reported to be enhanced in most plants under heavy metal toxicity, thereby protecting the cells from dehydration stress [8]. The enzymatic [superoxide dismutase (SOD), ascorbate peroxidase (APX), catalase (CAT), glutathione reductase (GR), monodehydroascorbate reductase (MDHAR) and dehydroascorbate reductase (DHAR)] as well as non-enzymatic antioxidants [ascorbic acid (AsA), glutathione (GSH), α-tocopherols and phenolic compounds] can effectively diminish the production of ROS [12,13]. For the detoxification of MG, plants possess the glyoxalase system consisting of glyoxalase I (GlyI) and glyoxalase II (GlyII) which regulates the production of MG. Plants make the full use of their defense system under stress, however, external application of some agents like mineral elements, chemicals, etc. could strengthen the defense system. Hence, the use of nanoparticles provides a new and sustainable approach to mitigate As stress.

In recent times, the use of micro- and macronutrients in nanoparticles (NPs) form has been considered as an effective approach for improving the growth and production of most crops [14]. These nanoparticle supplementations may help to reduce the loss of nutrients and enhance crop production in a sustainable manner [15]. Among metal-based NPs, there is an increasing interest in the application of zinc oxide (ZnO) NPs in agricultural sciences [15,16]. Still, very few studies have reported the impact of ZnO–NPs in the soil-plant system. The plant species, type of the soil and soil pH are the main components which influence the availability of Zn in soils and ZnO–NPs toxicity in plants [17]. Interestingly, low availability of Zn in soils possibly supports the foliar application of ZnO–NPs. Many studies have reported nanoparticle formulations of zinc and their application as a foliar spray to be efficient in decreasing the accumulation of heavy metals in plants [18]. However, at higher concentration, ZnO–NPs may prove to be toxic for plants and act as an obstacle for using it as a nano-fertilizer, but at the same time, at a lower concentration it may prove to be favorable for plants depending upon their growth environment and types of species [19,20]. This study was thus designed to explore the effect of ZnO–NPs application on As-stressed soybean plants by elucidating its impact on various growth and metabolic factors.

## 2. Results

### 2.1. Effect of ZnO–NP and As on the Lengths of Shoot and Root and Biomass Yield

**Figure 1:**
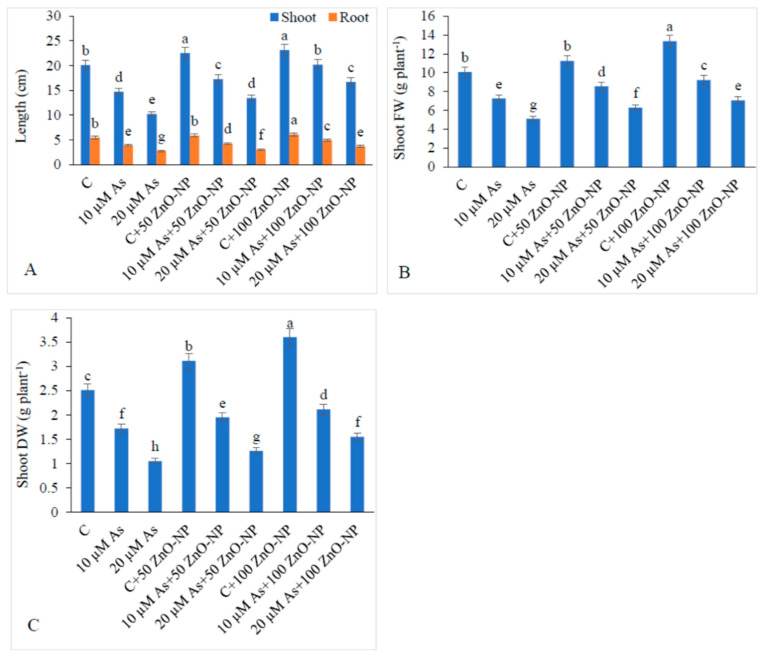
Effect of different concentrations of As and ZnO–NP on (**A**) shoot and root length, (**B**) shoot fresh weight and (**C**) shoot dry weight in soybean plants. Different letters indicate significant difference between the treatments. The data are the means ± SE (*n* = 5).

Shoot length declined by 26.76% and 49.0% at 10-µM- and 20-µM-As concentration, respectively, with respect to the control (non-arsenic-treated plants). Application of 50-mg/L ZnO–NP on the plants increased the shoot length by 17.59% and 31.12% under 10-μM- and 20-μM-As stress, respectively, over the controls (only As-treated plants). Supplementation of ZnO–NP (100 mg/L) enhanced the shoot length by 37.36% and 63.02% with 10- and 20-μM-As stress, respectively, relative to the controls (only-As-treated plants) (Figure 1A).

Similarly, in case of roots, at zero concentration of ZnO–NP (0 mg/L), the root length declined by 39.27% and 49.26% at 10-µM and 20-µM As, respectively, over the controls (non-As-treated plants). Application of 50-mg/L ZnO–NP on the plants increased the root length by 8.14% and 10.10% under 10 and 20-μM-As stress, respectively, with respect to the controls (non-As-treated plants). Application of ZnO–NP (100 mg/L) enhanced the root length by 25.44% and 33.93% under 10- and 20-μM-As stress, respectively, relative to the plants treated with As only and 50-mg/L ZnO–NP-treated plants (Figure 1A).

Arsenic stress decreased the shoot fresh and dry weights (Figure 1B,C). Shoot dry weight (DW) declined by 31.47% and 58.16%, with 10-μM- and 20-μM-As stress, respectively, with reference to those in the non-As-treated plants. Application of 50-mg/L ZnO–NP to 10-μM- and 20-µM-As-treated plants enhanced the shoot DW by 13.37% and 19.99%, respectively, relative to those in the plants treated with As alone. However, application of 100-mg/L ZnO–NP to 10-μM- and 20-µM-As-treated plants enhanced the shoot DW by 22.67% and 47.61%, respectively, over those in the plants treated with As alone (Figure 1C).

### 2.2. Effect of As and ZnO–NP on Pigment Content

**Figure 2:**
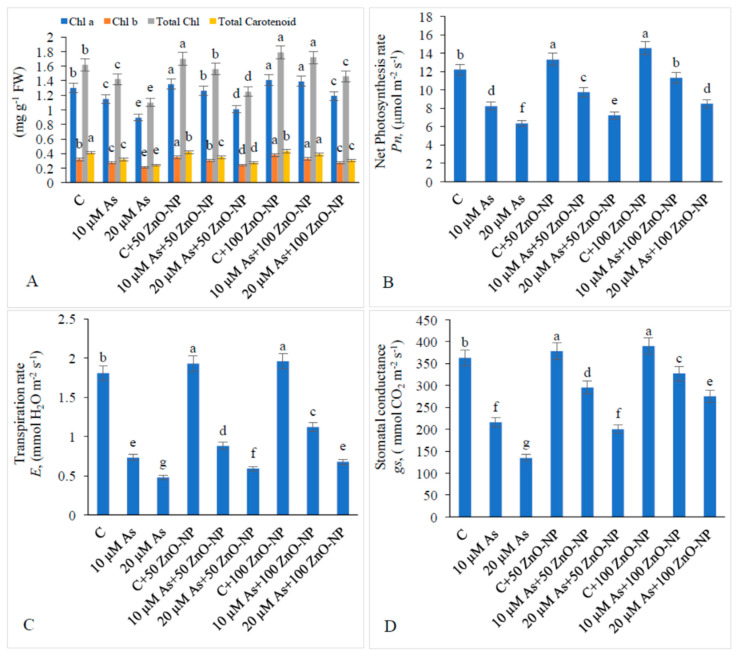
Effect of different concentrations of As and ZnO–NP on (**A**) pigment content, (**B**) net photosynthesis rate, (**C**) transpiration rate and (**D**) stomatal conductance in soybean plants. Different letters indicate significant difference between the treatments. The data are the means ± SE (*n* = 5).

Supplementation of 10-μM As decreased the amount of total chlorophyll and carotenoids by 12.34% and 21.95%, respectively. Similarly, supplementation of 20-μM As decreased the amount of total chlorophyll by 32.09% and carotenoids by 41.46% relative to the those in the non-As-treated plants. Application of 25-mg/L ZnO–NP to 20-μM-As-stressed plants enhanced the total chlorophyll by 13.63% and carotenoids by 12.50% over those in the plants treated with As only. Application 100-mg/L ZnO–NP to 20-μM-As-stressed plants enhanced the total chlorophyll by 32.72% and carotenoids by 25% with respect to those in the plants treated with As only (Figure 2A).

### 2.3. Effect of As and ZnO–NP on Gas Exchange Attributes

Gas exchange parameters i.e., net photosynthesis rate (*Pn)*, transpiration rate (*E*) and stomatal conductance (*g_s_)* declined significantly with an increase in As stress. Supplementation of 50-mg/L ZnO–NP to 10-μM-As-stressed plants enhanced *Pn by* 18.18%, *E by* 20.54% and *g_s_* by 36.57% and that to 20-μM-treated plants’ *Pn by* 13.70%, *E by* 22.91% and *g_s_ by* 48.14%. Application of 100-mg/L ZnO–NP to 20-μM-As-stressed plants enhanced *Pn, E* and *g_s_ by* 34.01%, 41.66% and 103.70%, respectively, over those in the As-alone-stressed plants (Figure 2B–D).

### 2.4. Effect of As and ZnO–NP on Chlorophyll Fluorescence

**Figure 3:**
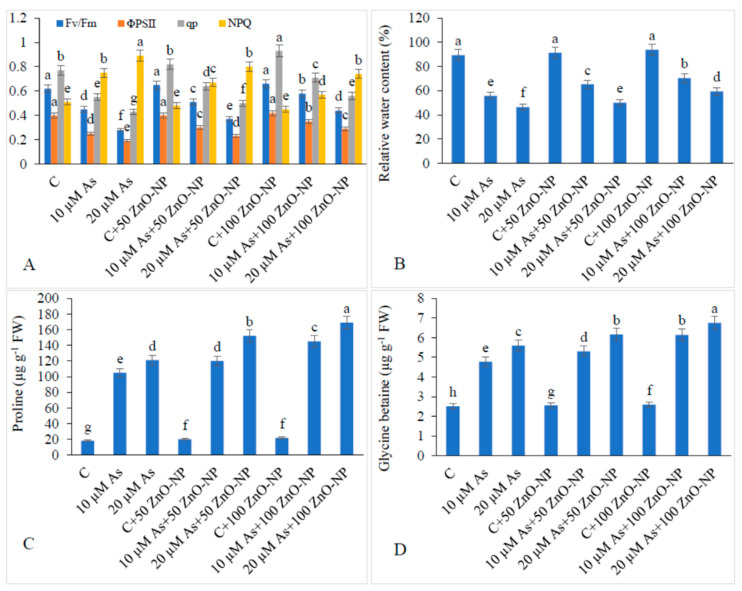
Effect of different concentrations of As and ZnO–NP on (**A**) chlorophyll fluorescence, (**B**) relative water content, (**C**) proline content and (**D**) glycine betaine content in soybean plants. Different letters indicate significant difference between the treatments. The data are the means ± SE (*n* = 5).

The maximum quantum efficiency (*F*v/*F*m) and photochemical yield (ΦPSII) of photosystem II declined with an increase in As stress than those in the non-As-treated plants. When 10-μM-As-stressed plants were supplemented with 50-mg/L and 100-mg/L ZnO–NP, both *F*v/*F*m and ΦPSII increased by 13.33%, 19.99% and 32.14%, 21.05%, respectively, with respect to those in the As-alone-treated plants. In addition, when 20-μM-As-stressed plants were supplemented with 50-mg/L and 100-mg/L ZnO–NP, both *F*v/*F*m and ΦPSII further increased by 28.88% and 39.99% and 52.63% and 57.14%, respectively, relative to those in the As-alone-treated plants (Figure 3A).

The photochemical quenching (qp) declined in the As-stressed plants and non-photochemical quenching (NPQ) increased in the As-stressed plants with respect to that in the non-As-fed plants (Figure 3A). Application of 50-mg/L and 100-mg/L ZnO–NP increased the qp by 16.27% and 30.23% in the 20-μM-As-stressed plants. In addition, application of 50-mg/L and 100-mg/L ZnO–NP declined the NPQ by 10.11% and 15.73% in the 20-μM-As-stressed plants. (Figure 3A).

### 2.5. Effect of As and ZnO–NP on Relative Water Content (RWC)

Leaf relative water content (RWC) decreased by 37.71% and 48.07% in the As-stressed plants with reference to that in the non-As-treated plants. However, the supplementation of 50-mg/L and 100-mg/L ZnO–NP to the 10-μM-As-stressed plants increased the water content by 17.14% and 26.26%, respectively, over those in the As-alone-treated plants. In addition, supplementation of 50-mg/L and 100-mg/L ZnO–NP to the 20-μM-As-stressed plants further increased the RWC by 7.87% and 27.79% (Figure 3B).

### 2.6. Appraisal of Proline and Glycine Betaine (GB) by ZnO–NP in As-Treated Plants

The amount of proline increased by 478.51% and 566.66%, in 10- and 20-μM-As-stressed plants, respectively, over that in the non-As-treated plants. Supplementation of 50-mg/L and 100-mg/L ZnO–NP further increased the proline content by 14.28% and 38.09%, respectively, in the plants exposed to 10 and 20-μM As. In addition, when the 20-μM-As-stressed plants were treated with 50-mg/L and 100-mg/L ZnO–NP, the proline content further increased by 25.61% and 39.66%, respectively, compared to those in the plants treated with As only (Figure 3C).

The amount of glycine betaine (GB) increased by 90.43% and 122.70%, in the 10- and 20-μM-As-stressed plants, respectively, with respect to that in the non-As-fed plants. Supplementation of 50-mg/L and 100-mg/L ZnO–NP further increased the GB content by 11.08% and 28.66% in the plants exposed to 10- and 20-μM As. Moreover, when the 20-μM-As-stressed plants were treated with 50-mg/L and 100-mg/L ZnO–NP, the GB content increased by 10.19% and 20.75% (Figure 3D).

### 2.7. Measurement of H_2_O_2_, Lipid Peroxidation (MDA) and Electrolyte Leakage (EL)

**Figure 4:**
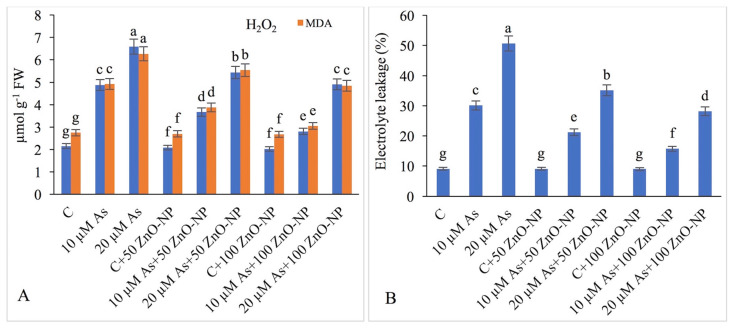
Effect of different concentrations of As and ZnO–NP on (**A**) accumulation of H_2_O_2_ and MDA content and (**B**) electrolyte leakage in soybean plants. Different letters indicate significant difference between the treatments. The data are the means ± SE (*n* = 5).

The amount of H_2_O_2_ increased by 126.97% and 206.51%, in the 10- and 20-μM-As-stressed plants, respectively, with respect to that in the non-As-treated plants. However, exogenous application of 50-mg/L ZnO–NP enhanced the H_2_O_2_ content by 70.69% and of 100-mg/L ZnO–NP by 30.69% only in the 10-μM-As-stressed plants as compared to the respective controls. In plants treated with 20-μM As and additionally supplied with 50-mg/L and 100-mg/L ZnO–NP, the H_2_O_2_ content increased by 153.02% and 128.37%, respectively, compared with those of As-treated plants which received no ZnO–NP (Figure 4A).

The MDA concentration increased by 126.97% and 206.51%, in the 10- and 20-μM-As-stressed plants, respectively, over those in the non-As-treated plants. The MDA content decreased by 21.13% in the 50-mg/L ZnO–NP-treated plants and 11.64% in the 100-mg/L ZnO–NP-treated plants under 10-μM As. The plants treated with 20-μM As when supplied with 50-mg/L and 100-mg/L ZnO–NP, the MDA concentration further declined by 38.0% and 22.64%, respectively, compared with those in the plants treated with As only (Figure 4A).

The As-stressed 10- and 20-μM plants showed increased electrolyte leakage by 230.98% and 456.81%, respectively relative to that in the non-As-treated plants. Exogenous application of 50-mg/L and 100-mg/L ZnO–NP declined the electrolyte leakage by 29.54% and 30.68%, respectively, in the 10-μM-As-stressed plants compared to those in the plants treated with As only. The 20-μM-As-stressed plants when provided ZnO–NP, showed reduced electrolyte leakage by 47.80% with 50-mg/L ZnO–NP and 44.36% with 100-mg/L ZnO–NP over those in the As-only-treated plants (Figure 4B).

### 2.8. Antioxidant Enzyme Activities and Ascorbate–Glutathione Cycle

**Figure 5:**
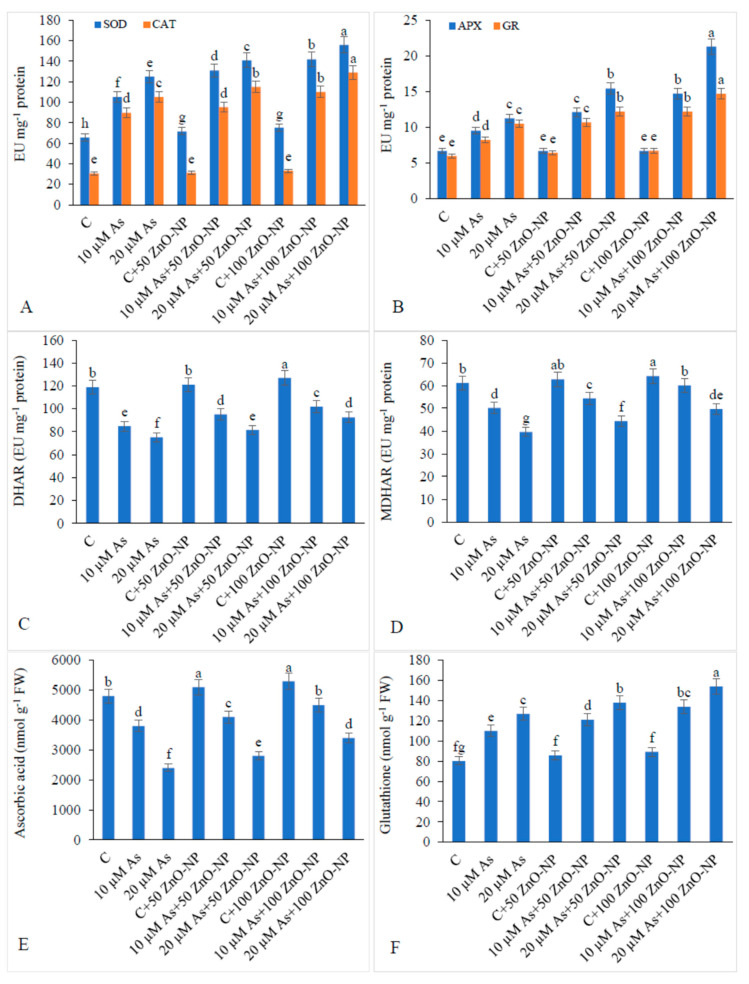
Effect of different concentrations of As and ZnO–NP on activity of (**A**) superoxide dismutase (SOD) and catalase (CAT), (**B**) ascorbate peroxidase (APX) and glutathione reductase (GR), (**C**) dehydroascorbate reductase (DHAR), (**D**) monodehydroascorbate reductase (MDHAR), (**E**) ascorbic acid and (**F**) glutathione content in soybean plants. Different letters indicate significant difference between the treatments. Data are means ± SE (*n* = 5).

The activities of SOD and CAT were found to be enhanced under 20-μM-As stress by 90.69% and 243.69%, respectively, over those in the non-As-fed plants. The activities of these enzymes were further enhanced when the 20-μM-treated plants were supplemented with 50-mg/L ZnO–NP (12.8% and 9.52%) and 100-mg/L ZnO–NP (24.8% and 22.85%) (Figure 5A).

The activities of the Asc–Glu cycle enzymes, APX and GR, increased under 20-μM-As stress by 90.69% and 243.69%, respectively, compared to those in the non-As-treated plants. The activities of these enzymes were further enhanced by 37.17% and 16.27%, respectively, in the 20-μM-As plants when they were supplemented with 50-mg/L ZnO–NP compared to those in the plants exposed to As only. The activities were further increased by 89.17% and 39.96%, respectively, with 100-mg/L ZnO–NP in the 20-μM-As-stressed plants (Figure 5B).

The activities of DHAR and MDHAR were found to be declined under 20-μM-As stress by 36.78% and 35.38%, respectively, compared to those of the non-As-treated plants. However, the activities of these enzymes increased markedly (8.29% and 12.25% relative to the controls) when supplemented with 50-mg/L ZnO–NP. On supplementation of 100-mg/L ZnO–NP to the 20-μM-As-stressed plants, the activity of DHAR further increased by 23.0% and that of MDHAR by 25.68% when compared with those of the plants exposed to As only (Figure 5C,D).

The amount of ascorbic acid (AsA) declined by 50% with 10-μM As and 20.83% by 20-μM-As stress over that in the non-As-treated plants. However, the levels of AsA in the As-stressed plants were found to be enhanced when they were supplemented with 50-mg/L and 100-mg/L ZnO–NP (Figure 5E).

The amount of glutathione (GSH) increased under 10- and 20-μM-As stress by 37.24% and 58.45%, respectively, compared to that in the non-As-treated plants. However, enhancement in the levels of GSH was observed with 50-mg/L and 100-mg/L ZnO–NP by 8.66% and 21.25%, respectively in the plants treated with 20-μM As compared to that in the plants exposed to As only (Figure 5F).

### 2.9. Methyl Glyoxal (MG) and Glyoxalase (Gly) System in As-Stressed Plants

**Figure 6:**
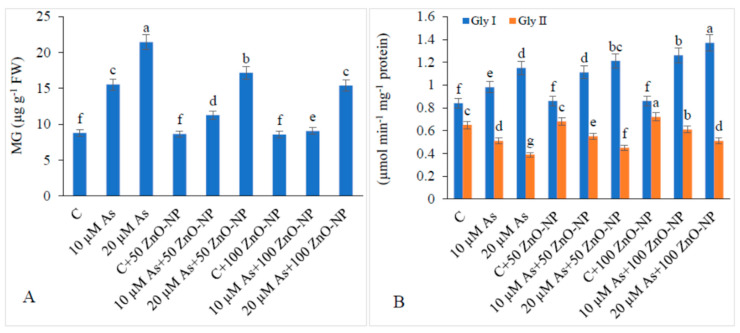
Effect of different concentrations of As and ZnO–NP on (**A**) MG content and (**B**) activity of GlyI and GlyII in soybean plants. Different letters indicate significant difference between the treatments. Fata are means ± SE (*n* = 5).

The As concentration of 10-μM and 20-μM increased the MG content by 76.90% and 144.02%, respectively. However, the levels of MG declined when 20-μM-As-stressed plants were supplemented with 50-mg/L (19.99%) and 100-mg/L ZnO–NP (28.15%) with respect to those in the plants exposed to As only (Figure 6A).

Application of 10-μM and 20-μM As stress increased the activity of GlyI by 16.66% and 36.90%, however, GlyII decreased by 21.53% and 40%, respectively, relative to those in the non-As-treated plants. The activity of GlyI increased by 5.21% and 19.13% when 20-μM-As-stressed plants were supplemented with 50-mg/L and 100-mg/L ZnO–NP, respectively. The activity of GlyII also increased by 15.38% with 50-mg/L and 30.76% with 100-mg/L ZnO–NP in the plants treated with 20-μM As over those in the plants exposed to As only (Figure 6B).

### 2.10. Effect of ZnO–NP and As on Accumulation of As in Shoot and Root

The 10-μM and 20-μM-As-stressed plants showed shoot As 395 μg g^−1^ DW and 500 μg g^−1^ DW, respectively, however, supplementation of 50-mg/L ZnO–NP decreased As accumulation in the shoots to 250 μg g^−1^ DW under 10-μM As and to 370 μg g^−1^ DW under 20-μM As. Accumulation of As in the shoots was further decreased to 175 μg g^−1^ DW and 220 μg g^−1^ DW, when the 10-μM and 20-μM-As-stressed plants were supplied with 100-mg/L ZnO–NP (Table 1).

In roots, the accumulation of 500-μg g^−1^ DW As under 10-μM As and 700-μg g^−1^ DW As under 20-μM As was recorded in the present study. However, application of 50-mg/L ZnO–NP reduced the accumulation of As to 420-μg g^−1^ DW and 600-μg g^−1^ DW in the 10-μM- and 20-μM-As-stressed plants, respectively. The accumulation of As in the roots was further decreased to 370-μg g^−1^ DW with 10-μM + 100-mg/L ZnO–NP and 400 with 20-μM + 100-mg/L ZnO–NP (Table 1).

## 3. Discussion

ZnO nanoparticles release Zn which regulates growth as it is an important element involved in the synthesis/accumulation of auxin, indole-3-acetic acid, which plays a vital role in cell division and cell expansion (Ali and Mahmoud, 2013). According to Hafeez et al. (2013) Zn application maintains membrane stability through binding to sulfhydryl groups and phospholipids under stress conditions. For example, Weisany et al. (2014) reported that Zn is essentially required for maintaining the structural integrity of membranes in soybean under salinity stress. In fact, Zn application boosts the uptake of micro- and macronutrients which are declined under stress conditions (Abd El-Hady 2007; Weisany et al. 2014). Furthermore, Zn supplementation was found to be beneficial in restoring the uptake of nutrients like K, Mg, Ca, Fe, P, thereby maintaining the structural and functional ability of different organelles like chloroplast, mitochondrion, etc. (Ahmad et al., 2018). Zinc helps in the uptake of Mg, one of the main constituents of the chlorophyll molecule, which can restore photosynthesis. Zinc is also reported to reduce ROS-induced oxidative stress, which may be attributed to the enhanced activity of enzymatic antioxidants (Ahmad et al., 2018), especially SOD, which contains different metal ions like copper and zinc (Cu/ZnSOD), manganese (MnSOD) and iron (FeSOD). However, in contrast, deficiency of Zn may lead to a variety of dysfunctions in plants. For example, Zn deficiency led to membrane disorganization in the thylakoids of sugar beet leaves (Henriques 2001). In other studies, Cakmak and Marschner (1988a, 1988b) reported that cotton roots deficient in Zn had enhanced potassium (K) leakage and it could be mitigated by the exogenous supplementation of Zn.

Arsenic toxicity affected the growth of soybean plants in terms of shoot length and shoot and root fresh and dry weights. These results endorse the findings of Jung, et al. [21] in As-stressed rice seedlings. Abedin, et al. [22] have also demonstrated decreased growth in rice due to As toxicity. Arsenic is believed to react with thiol groups of enzymes leading to overall inhibition of metabolism. Arsenic mediated growth inhibition was suggested to be attributable to cell cycle arrest and inhibition of DNA synthesis and repair mechanisms [23]. Many other heavy metals like cadmium, mercury, lead and chromium—as well as As—have also been shown to cause decreased growth and biomass yield in different plants [7,16,24,25]. This decreased growth and biomass yield of plants exposed to heavy metal stress may be due to restricted uptake of nutrient elements, reduced photosynthesis and other metabolic activities [7]. However, the supplementation of ZnO–NP in our study enhanced the growth and biomass yield of As-stressed soybean plants significantly. These results coincide with the findings of Rizwan, et al. [16], who also reported significantly increased growth of Cd-stressed maize plants with the application of ZnO–NP. ZnO–NPs have also been shown to enhance the growth and biomass yield of plants under Cd and Pb stress [26]. An interplay of various factors such as increase in mineral uptake, decrease in oxidative stress, triggering of biochemical pathways involved in biomass accumulation, etc., may play a significant role in such growth enhancement [26,27]. ZnO–NP application has been reported to decrease the oxidative stress induced by Cd and Pb in many other plants including *Leucaena leucocephala* [26]. Additionally, we also found that As hampered the growth of roots more than that of the shoots, possibly because retention of As in the roots was higher than that in the stem. Several studies have reported the accumulation of As being higher in roots than that in stem [5,28]. In contrast, some studies have also reported that the effect of As on root and shoot growth may vary depending on plant species, contamination level and plant tissue ability to uptake As [26].

All the photosynthetic parameters of the soybean plants were observed to be diminished under As stress. Arsenic is known to interfere with nitrogen metabolism leading to its reduced bioavailability [29]. Nitrogen is a core component of chlorophyll, so its reduced bioavailability due to arsenic stress diminishes the chlorophyll content of a plant [29]. This could be one of the main reasons for the decreased photosynthetic rate of plants in the presence of arsenic [29]. Our findings of reduced photosynthetic pigments and photosynthetic rate are in line with some previous studies on a variety of other plants exposed to arsenic stress [5,14]. Application of ZnO–NP considerably increased the amount of total chlorophyll in leaves, and it also had a positive effect on gas exchange characteristics including net assimilation rate, transpiration and stomatal conductance. These results endorse the findings of Faizan, et al. [30] which showed improved photosynthetic rates in rice plants on application of ZnO–NP. This may have been due to the ability of metal nanoparticles to enhance the production of chemical energy in photosynthetic systems, synthesis of photosynthetic pigments and improvement in quantum yield in plants [27,31,32,33]. Some earlier studies have highlighted the efficiency of NPs in enhancing photosynthesis in plants whose response varies in a dose-dependent and plant species-dependent manner [14,34]. Furthermore, our study showed that leaf relative water content (RWC) also decreased in the presence of As in the soybean plants. Similar results have been reported in other studies [35]. However, when arsenic-stressed soybean plants were supplemented with different concentrations of ZnO-NP, there was a considerable increase in water potential and RWC of the leaves. This may have been due to enhanced uptake of water and mineral elements in the presence of ZnO–NP.

Arsenic stress increased the content of essential osmolytes particularly of proline and glycinebetaine in the present study. These osmolytes are important in alleviating the stress-mediated damaging effects. Thus, an increase in their content under toxic conditions is a natural way of defense in plants [36,37]. Arsenic stress enhanced the proline accumulation in the soybean plants as has been reported by Choudhury, et al. [38] in *Oryza sativa* and Siddiqui, et al. [39] in *Withania somnifera*. The enhanced accumulation of proline and GB protects biochemical processes, promotes ROS scavenging, and maintains redox homeostasis and functions of different enzymes [40]. According to Hasanuzzaman, et al. [41], proline and GB help maintain the plant antioxidant and glyoxalase systems thereby enhancing tolerance against stress. Anjum, et al. [42] showed that GB and GSH help in metal chelation that reduces the noxious effects of metal stress. To decrease the stress or enhance the stress withstanding potential of plants, naturally occurring mechanisms could be made stronger by supplementation of agents such as metal oxides which can boost the antioxidant and glyoxalase systems. Thus, the use of ZnO–NP helps alleviate heavy metal toxicity, as has been found in this study. These findings are in line with other studies [18,43]). According to Sivakumar, et al. [44] the carboxylase activity of rubisco was protected by enhanced proline content which in turn impeded photoinhibition. An increase in photosynthesis under stress conditions correlates with the accumulation of GB [45]. ZnO–NP seems to upregulate the proline and GB biosynthetic pathways so as to attain maximum accumulation of these two osmolytes in plants, which could play a vital role in alleviation of As stress.

The increasing intensity of As stress in the soybean plants caused increased levels of H_2_O_2_ and MDA and electrolyte leakage (EL). Enhanced accumulation of H_2_O_2_ and MDA as well as increased EL has also been reported in other plants grown under different stresses ((Ahmad et al., 2018, 2020; Ahanger and Agarwal 2017 a,b; Kaya et al., 2020). The H_2_O_2_ accumulation needs to be maintained to a certain threshold level, otherwise it will lead to lipid peroxidation that in turn leads to electrolyte leakage. Supplementation of ZnO–NP in our study decreased the accumulation of H_2_O_2_ and MDA as well as EL in the present study, and our results coincide with the findings of some earlier reports with different plant species [16,18,34]. Enhanced activity of antioxidant enzymes by supplementation of ZnO–NPs may scavenge H_2_O_2_ and elevate mineral uptake, thereby reducing plant oxidative stress [16].

Plants are known to exhibit several detoxification mechanisms such as activation of different antioxidant enzymes such as superoxide dismutase (SOD), ascorbate peroxidase (APX) and catalase (CAT) and non-enzymatic antioxidants like glutathione, ascorbate, tocopherols, etc. against enhanced ROS accumulation [13,46]. Arsenic stress-induced enhanced activities of antioxidant enzymes corroborate with the findings of Talukdar [47], Kumar Yadav and Srivastava [48] and Siddiqui, et al. [49] in *Trigonella goenum-graecum*, *Zea mays* and *Ocimum tenuiflorum,* respectively. Lu, et al. [50] also reported enhanced activities of SOD, CAT, POD and APX in tartary buckwheat under Cd stress. Arsenic stress also decreased the components involved in the Asc–Glu cycle [50]. The activities of DHAR and MDHAR as well as the levels of AsA showed a decline with As toxicity in the present study and these results are in line with our earlier findings on *Vicia faba* [8]. Glutathione reductase (GR) has been reported as a rate limiting enzyme in the Asc–GSH cycle and it catalyzes the reaction of GSSG (oxidized form of GSH) to GSH with the electron donor NADPH [51,52]. The DHAR enzyme catalyzes the reduction of DHA to AsA [51,52]. According to Sharma [53] As binds to thiol groups of antioxidant enzymes which directly affect biochemical reactions thereby hampering overall plant growth. The enhanced activities of antioxidant enzymes against the oxidative stress have also been reported through the over-expression of SOD, APX and CAT isozymes [47]. Supplementation of As-stressed soybean plants with ZnO–NP further increased the activities of these defense enzymes, indicating a protective role of ZnO–NP in such adverse conditions. The ZnO–NP induced enhanced activities of SOD, CAT, APX and GR in the As-stressed soybean plants in the present study endorse the results of [26] in *Leucaena leucocephala* seedlings under Cd and Pb stresses. ZnO–NP-induced enhanced activities of SOD, CAT, APX and GR have been reported by many authors in different plants, e.g., Tripathi, et al. [43] in *Pisum sativum*, Hernandez-Viezcas, et al. [54] in *Prosopis juliflora*, Krishnaraj, et al. [55] in *Bacopa monnieri.* These enhanced activities of antioxidants helped the plants to scavenge the extra ROS produced under stressful cues.

Another detoxifying system known as the glyoxalase cycle which contains glyoxalase I and glyoxalase II (Gly I and Gly II) and their function is to remove the MG generated during stress [56]. MG accumulation under As stress is the main cause of cytotoxic and mutagenic effects and alteration of cellular ultrastructure and cell death [11]. However, Gly I and Gly II reduce the accumulation of MG as has been reported by earlier workers [8,57]. Supplementation of ZnO–NP enhanced the activity of Gly I and Gly II, thus providing an extra strength to minimize the production of MG. Little literature is available on the interaction of As and ZnO–NP in plants with respect to MG accumulation. Thus, further investigation at physiological and molecular levels needs to be carried out.

## 4. Materials and Methods

### 4.1. Plant Material and Experimental Design

Soybean seeds were decontaminated with sodium hypochlorite (0.5%, *v/v*) for 5 min and then soaked in distilled water for 10 min before germination. The germinated seeds (4-day-old seedlings) were shifted to pots containing vermicompost and sand (1:3 ratio) (sterilized) and kept for 3 weeks without any interruption. Thereafter, arsenic (AsIII, salt NaAsO_2_, sodium arsenite) was added to each pot daily for 5 weeks (60-day-old plants). AsIII was used in this study because it is more soluble and mobile than other forms of As. Only full-strength Hoagland’s nutrient solution was added to the experimental control. After 2 weeks of As treatment (39-day-old plants), three different concentrations (0, 50 and 100 mg L^−1^) of ZnO–NP (Sigma Aldrich, Saint Louis, USA) were sprayed to plant foliage every alternate day for 2 weeks (60-day-old plants). After adding ZnO–NP in the pots, they were maintained in a growth chamber with the following conditions: 26/15 °C (day/night) temperature, 18 h/6 h (light/dark) photoperiod and 70%–75% relative humidity. Each treatment had five replications and each experiment was repeated 3 times. After growing the plants for 60-days, they were harvested and studied for different parameters.

### 4.2. Estimation of Length and Dry Mass of Shoot and Root

Shoot and root lengths were measured using a manual scale after uprooting the plants. The samples were measured for fresh weight and then subjected to an oven for drying at 70 °C for 24 h for dry weight estimation.

### 4.3. Evaluation of Photosynthetic Pigments

Leaf samples of 300 mg each were crushed with 80% acetone. The mixtures were centrifuged for 20 min at 3000× *g*. The optical density (OD) of the supernatant was estimated with a spectrophotometer at 480, 645 and 663 nm following Arnon [58].

### 4.4. Leaf Gas Exchange Parameters and Chlorophyll Fluorescence (Fv/Fm, ΦPSII, qP, NPQ)

Leaf gas exchange parameters including transpiration rate (*E*), net photosynthetic rate (*Pn*) and stomatal conductance (*g_s_*) were estimated using the IRGA (LCA-4 model Analytical Development Company, Hoddesdon, UK) using fully expanded leaves from each replicate between 10:00 h and 12:00 h in full and bright sunlight.

For the determination of chlorophyll fluorescence parameters, Junior PAM Chlorophyll Fluorometer (H. Walz, Effeltrich, Germany) was used following the protocol of Li, et al. [59].

### 4.5. Determination of Leaf (LRWC)

For the assessment of relative water content of leaves, the method of Smart and Bingham [60] was used and its values were determined with the following formula:

(1) $$\mathrm{LRWC}=\frac{\mathrm{FW}-\mathrm{DW}}{\mathrm{TW}-\mathrm{DW}}\times \;100$$

### 4.6. Determination of Proline and Glycine Betaine (GB) Content

For determining the proline content, the protocol given by Bates, et al. [61] was used and the optical density (OD) was recorded at 520 nm with a spectrophotometer. For determining the glycine betaine (GB) content, the Grieve and Grattan [62] method was used, and the absorbance was taken at 365 nm.

### 4.7. Measurement of H_2_O_2_, Lipid Peroxidation (MDA) and Electrolyte Leakage (EL)

For measuring the H_2_O_2_ and MDA contents, the methods given by Velikova, et al. [63] and Madhava Rao and Sresty [64], respectively, were used. The OD was recorded at 390 nm for H_2_O_2_ and at 432 and 600 nm for MDA with a spectrophotometer. The method of Dionisio-Sese and Tobita [65] was used for measuring the electrolyte leakage, and its values were determined using the following formula:

(2) $$\mathrm{EL}\%=\frac{\mathrm{EC}1-\mathrm{EC}0}{\mathrm{EC}2-\mathrm{EC}0}\times \;100$$

### 4.8. Estimation of Activities of Antioxidant Enzymes and the Ascorbate–Glutathione Cycle

Fresh leaf sample (each 500 mg) was crushed in polyvinyl pyrrolidone solution (1%) and cold potassium phosphate buffer (pH 7.0, 100 mM) for extracting the antioxidant enzymes. The mixture was centrifuged at 4 °C at 12,000 *g* for 30 min. The supernatant was collected and used for determining the activities of SOD, CAT, APX and GR.

#### 4.8.1. Superoxide Dismutase (SOD, EC1.15.1.1) Activity

The activity of SOD was determined by monitoring the diminishing of NBT (nitro blue tetrazolium) photochemically by the enzyme extract [66]. The OD was recorded at 560 nm and the SOD activity was presented as EU mg^-1^ protein.

#### 4.8.2. Catalase (CAT, EC1.11.1.6) Activity

The CAT activity was evaluated by the protocol given by Aebi [67] and disappearance of H_2_O_2_ was recorded for 2 min at 240 nm. The CAT activity was presented as EU mg^-1^ protein.

#### 4.8.3. Ascorbate Peroxidase (APX, EC1.11.1.1) Activity

APX activity was assayed by monitoring the H_2_O_2_-dependent oxidation of ascorbate at 290 nm for 2 min (Nakano and Asada 1981). The APX activity was presented as EU mg^−1^ protein.

#### 4.8.4. Glutathione Reductase (GR, EC1.6.4.2) Activity

The GR activity was assayed by the procedure described by Foster and Hess [68]. The absorbance was taken at 340 nm for 3 min and the GR activity was presented as EU mg^−1^ protein.

#### 4.8.5. Monodehydroascorbate Reductase (MDHAR, EC1.6.5.4) Activity

The protocol described by Miyake and Asada [69] was employed for the assay of MDHAR. The OD was recorded at 340 nm and the activity was presented as µmol NADPH oxidized (EU mg^−1^ protein).

#### 4.8.6. Dehydroascorbate Reductase (DHAR, EC1.8.5.1) Activity

Nakano and Asada [70] protocol was exercised for the assay of DHAR activity and the OD was taken at 265 nm. The DHAR activity was presented as EU mg^−1^ protein.

#### 4.8.7. Ascorbic Acid (AsA) and Glutathione

The methods described by Huang, et al. [71] and Yu, et al. [72] were followed for the assay of ascorbate and glutathione content, respectively.

### 4.9. Estimation of Methylglyoxal (MG), Gly I (EC4.4.1.5) and GlyII (EC3.1.2.6)

The protocol of Wild, et al. [73] was used for the determination of MG content and the OD was taken at 288 nm. For determining the activities of Gly 1 and Gly II, the protocols given by Hossain, et al. [74] and Mostofa and Fujita [75], respectively, were used. The OD was recorded at 240 nm and the enzymes’ activities were expressed as µmol mg^−1^ min^−1^ protein.

### 4.10. Estimation of As in the Shoot and Root

The dry tissue (each 500 mg) was digested in an acid mixture and the concentration of As was quantified by an atomic absorption spectrophotometer (Perkin-Elmer Analyst Model 300, NJ, USA).

### 4.11. Statistical Analysis

The SPSS statistical software version 17.0 (SPSS, Inc., Chicago, USA) was used for analysis of variance of each data set and the mean values were compared using Duncan’s multiple range test (DMRT) at *p* ≤ 0.05. The data presented are mean± SE and *n*= 5.

## 5. Conclusions

This study marks that ZnO–NPs are efficient ameliorators of As toxicity in soybean plants at different concentrations. Thus, if used at certain specific doses, ZnO–NPs could prove to be an excellent foliar fertilizer in areas that are prone to arsenic exposure. The use of these nanoparticles can increase the efficiency of cropping systems by mitigating As stress and promoting the yield of plants.

## Figures and Tables

**Table 1:** Effect of different concentrations of ZnO–NP on As accumulation in the leaf and root of soybean plants grown under As toxicity. Data presented are the means ± SE (*n* = 5). Different letters with mean values indicate significant difference at *p* ≤ 0.05. (ND, not detected).

ZnO–NP (mg L^−1^)	As (µM)	Shoot As (µg g^−1^ DW)	Root As (µg g^−1^ DW)
0	0	ND	ND
	10	395 ± 25.45b	500 ± 33.25c
	20	500 ± 32.56a	700 ± 42.11a
50	0	ND	ND
	10	250 ± 15.77d	420 ± 29.67d
	20	370 ± 20.89c	600 ± 45.55b
100	0	ND	ND
	10	175 ± 10.23f	370 ± 21.17f
	20	220 ± 13.77e	400 ± 26.53e
